# Perception of potential harm and benefits of HIV vaccine trial participation: A qualitative study from urban Tanzania

**DOI:** 10.1371/journal.pone.0224831

**Published:** 2019-11-08

**Authors:** Edith A. M. Tarimo, Joel Ambikile, Patricia Munseri, Muhammad Bakari

**Affiliations:** 1 Department of Nursing Management, Muhimbili University of Health and Allied Sciences, Dar es Salaam, Tanzania; 2 Department of Clinical Nursing, Muhimbili University of Health and Allied Sciences, Dar es Salaam, Tanzania; 3 Department of Internal Medicine, Muhimbili University of Health and Allied Sciences, Dar es Salaam, Tanzania; University of North Carolina at Chapel Hill, UNITED STATES

## Abstract

**Background:**

The development of an effective preventive HIV vaccine is the best-known option to halt incident HIV infections. Participants in HIV vaccine trials may possess expectations shaped by existing socio-cultural contexts that are important to understand to allow for improved trial design. Here, we describe post-phase I/II HIV vaccine trial perceptions within participating communities in Dar es Salaam, Tanzania.

**Materials and methods:**

This descriptive qualitative study was conducted in May 2016. We conducted eight focus group discussions, each consisting of 5 to 12 participants. Four groups comprised of the past phase I/II HIV vaccine trial participants and four groups involved those who did not participate. We used a thematic analysis approach.

**Results:**

Ongoing concerns existed among non-vaccine trial participants who believed that those who participated in HIV vaccine trials were infected with HIV. Limited post-HIV vaccine trial result dissemination, the pre-existing negative beliefs about vaccines, and experiences from other previous medical experiments fueled these concerns. The participants anticipated that broader dissemination of facts regarding HIV vaccine trials using media, former volunteers, and flyers would reduce the reported concerns. In contrast, some participants embraced the benefits gained through participating in HIV vaccine trials. HIV vaccine trial participants appreciated trial interventions, such as health status check-ups, knowledge acquisition, and facilitation of access to medical services. They envisioned mutual benefits in the form of community protection and capacity building among the local scientists.

**Conclusions:**

The future conduct of HIV vaccine trials in Tanzania requires wider community dissemination of information and post-trial feedback to alleviate concerns among the participating communities. Interventions such as medical services may represent essential incentives to the HIV vaccine trial volunteers. In future HIV vaccine trials, it is crucial to boost individual and perceived mutual benefits.

## Introduction

The search for an effective preventive HIV vaccine is underway [1] to address continuing incident HIV infections in regard to the public health agenda. HIV vaccine development continues, however, within historical, social, and cultural environments that may not easily support the performance of clinical trials and the eventual utilization of vaccines. Deep-rooted philosophical, political, and spiritual beliefs may underlie the observed opposition to vaccinations. For instance, the resistance to mandatory vaccination, which began in the 1850s in the UK, occurred due to concern regarding the infringement of personal liberty and choice [2]. The anti-vaccination reaction has continued in the USA due to fear of imaginary side effects, and this, along with the help of media and politicians, has fueled anti-vaccination movements [3]. The opposition to vaccination has continued into the subsequent decades [4–5] under the premise that vaccination causes a new disease with unknown etiology, namely autism [6]. Rigorous scientific studies, however, have failed to identify a link between autism and vaccines [7–8].

In low-income countries, understanding the vaccination culture of an area, the local experience regarding the HIV epidemic, and the perceptions within the community may be essential for successful future HIV vaccine trials [9]. Thus, socio-behavioral studies in conjunction with HIV vaccine trials are crucial for overcoming these existing challenges. A previous study identified challenges in risk communication, including correcting erroneous assumptions regarding live virus vaccination, clear explanation concerning the potential risks, possible side effects and adverse events, and communicating the issues surrounding partial efficacy [10]. In South Africa, Lesh *et al*. [11] identified barriers to HIV vaccine participation, and these included barriers that were both abstract (fear of illness/death, information and association with HIV/AIDS) and concrete (monetary costs of participation, fear of being tested, negative family reactions, negative community reactions and mistrust of researchers). Limited knowledge regarding HIV vaccine trials has been a commonly reported factor that results in elevated concerns among the participating communities [12–16]. Community engagement appears to be critical for the smooth implementation of HIV vaccine trials [17]. Although community engagement is an ethical requirement, it does not assure buy-in and participation [18]. In contrast, communication appears to be an essential component of community engagement; however, such communication should involve tailored activities, materials, and the best channels by which to communicate [19].

A phase IIb HIV vaccine trial known as the STEP study was conducted in Australia, Canada, Dominican Republic, Haiti, Peru, and Puerto Rico to obtain more information concerning the safety of the vaccine product in humans and to learn if the product can prevent HIV infection [1]. This study was terminated, as it did not demonstrate a decrease in HIV acquisition among the trial participants [20]. A previous study revealed that after STEP study termination, some participants expressed mistrust of trial sponsors, ethical concerns, and gaps in post-trial dissemination of information [21]. Another follow-up of the STEP study revealed mixed opinions regarding the termination of the STEP study, and participants expressed a fear of HIV-induced infection and questioned the issue of informed consent. Despite this, some participants in this study expressed altruism and intention of doing good for the public through the STEP study [22]. These mixed opinions underscore the need for innovative strategies to disseminate information regarding HIV vaccine trials to the public. Also, a previous post-HIV vaccine trial multisite study conducted in South Africa, India, Thailand, and Canada revealed the difficulties of communicating critical scientific concepts to community stakeholders, and these difficulties were due in part to the complex technical vaccine language and a lack of research experience by these stakeholders. Mistrust from historical experiences of colonialism and exploitation emerged as a challenge related to HIV vaccine trials in these sites [23].

Tanzania is one of the sub-Saharan African countries actively participating in HIV vaccine studies. In Dar es Salaam, Tanzania, certain phase I/II HIV vaccine trials, specifically HIVIS 03, TaMoVac 0I, and TaMoVac II, were conducted between 2007 and 2012. All trials adhered to good clinical practices. An exploratory pre-phase I/II HIV vaccine study revealed specific concerns towards the experimental vaccine among potential volunteers, who demanded a detailed understanding of the trial conduct and the reasons it was being conducted [24]. During the trial, fear of the negative outcome of the experimental vaccine and resistance from significant others were cited as the main reasons for declining to enroll in the HIV vaccine trial among eligible volunteers [25]. Those who enrolled and stayed on until the end of the trial, however, conveyed that participation offered them both the opportunity and the ability to cope with the doubts from the surrounding community [26]. The prolonged follow up of the HIVIS-03 study participants revealed that volunteers were likely to face negative comments from relatives and colleagues after the trial; however, those comments decreased over time [27]. Another exploratory study among the significant others (people who were perceived important by the youth who took part in the TaMoVac trial) revealed that community members could be an integral part of the recruitment and retention of young people into HIV vaccine trials despite a lack of prior knowledge regarding the vaccine trial [28].

Overall, the HIVIS-03, TaMoVac-01, and TaMoVac-II Phase I/II HIV vaccine trials revealed the candidate vaccine to be safe and highly immunogenic [29–31], even at lower doses and with easier administration schedules. Despite the success of these trials, mixed opinions were verbally expressed five years later from the participating communities (Police Force, Prison Force and Youths from the general population) in Dar es Salaam. This study describes perceptions of the participating communities at five years post completion of Phase I/II HIV vaccine trials in Dar es Salaam, Tanzania.

## Materials and methods

### Ethics statement

We obtained ethical clearance for this study from the Institutional Review Board (IRB) of the Muhimbili University of Health and Allied Sciences (MUHAS), Ref.No.2015-01-15/AEC/Vol.IX/44. We also obtained a verbal research permit from higher authorities of the Police and Prisons forces and the officer in charge of the Infectious Disease Clinic (IDC).

We obtained written informed consent from all participants. Specifically, participants were informed of the study objectives and that their participation was voluntary, as there was no coercion for participation. Participants were aware that there were no direct personal benefits due to their participation; however, the results of the study would be beneficial to the entire community and for the future implementation of HIV vaccine trial studies.

### Study setting and population

The study was conducted in Dar es Salaam, Tanzania. Male and female members of the Police and Prison forces and a group of youths that were ≥18 years and were currently attending the IDC were included in the study. The majority of the study participants had been exposed to HIV/AIDS educational sessions that included basic facts about HIV/AIDS and the importance of participation in HIV vaccine trials.

### Study design, sampling, and sample size

A descriptive qualitative design was used to explore the perceptions of HIV vaccine trial participants and non-trial participants. Both categories of participants were included in the current study to explore their perceptions regarding the completed Phase I/II HIV vaccine trials in Tanzania. Purposeful sampling methods were used to recruit potential participants based on their availability during data collection. A sample size of 64 to 96 was estimated based on the minimum and a maximum number of participants per focus group discussion. This allowed for 8 to 12 participants per group for eight pre-determined groups.

### Data collection

We conducted a total of eight Focus Group Discussions (FGDs). Four FGDs were conducted that included police officers who took part in previous HIV vaccine trials [29–30] and officers who did not take part with different groups by gender. The non-HIV vaccine trial volunteers were identified by study collaborators from the respective communities (Police Force, Prison Force, and IDC). Each group was comprised of 5 to 12 participants. We conducted two FGDs that included prison officers who took part in an HIV vaccine trial and officers who did not, and the groups consisted of six participants each. Also, we conducted two FGDs that included youths who took part in an HIV vaccine trial and youths who did not take part, and the two groups consisted of six and eight participants, respectively. More FGDs were conducted using police officers than were conducted using others, as the police officers comprised the largest number of volunteers within the HIV vaccine trials. We used the FGD guide to explore the perceptions of the participants regarding the HIV vaccine trials (HIVIS-03, TaMoVac-01, and TaMoVac-II). The discussions were moderated, and a designated note taker took notes during the discussions. All FGDs were digitally audio-recorded with consent from the participants.

### Data analysis

We transcribed all FGDs verbatim. We analyzed the data using a thematic analysis approach that involved reading and re-reading the text, manual coding in the margins, and grouping data in relatively exhaustive codes [32]. The two authors, EAMT and JSA, independently analyzed the data in the original language (Kiswahili) to minimize the possibility of losing the original meaning of concepts. We used thematic analysis to identify patterns of themes in the FGD data. We presented the preliminary findings to all authors, and we discussed the analysis process. Minor discrepancies were discussed and agreed upon. The discussion of preliminary findings and feedback from all authors ensured adequacy of analysis and emerged themes. Translation into English was performed for all data that were included in the article. Quotes were added to the manuscript to reflect the intended meaning of participants.

## Results

### Socio-demographic characteristics

A total of 67 participants took part in this study, and their mean age (±SD) was 31 (±7.1) years. Of the 67 participants, 36 were males. The highest level of education for most participants (70.1%, n = 47) was secondary education, while 15 (22.4%) and 5 (7.5%) completed seven years of primary and college education, respectively. Twenty-nine were former volunteers in HIV vaccine trials. Table 1 provides the FGD composition.

**Table 1:** FGD composition.

FGD No.	FGD type	Sex	No of participants
1	Police Officers HIV vaccine trial participants	Male	12
2	Police Officers HIV vaccine trial participants	Female	5
3	Prisons Officers HIV vaccine trial participants	Male	6
4	Youth HIV vaccine trial participants	Female	6
5	Police Officers non-HIV vaccine trial participants	Male	12
6	Police Officers non-HIV vaccine trial participants	Female	12
7	Prison Officers non-HIV vaccine trial participants	Male	6
8	Youth non-HIV vaccine trial participants	Female	8

The findings revealed two themes, specifically “**Concerns regarding vaccine-induced infection**” and “**Potential benefits of participation in HIV vaccine trials**." These themes are supported by five sub-themes and 12 codes as detailed below (Table 2).

**Table 2:** Themes, sub-themes, and codes.

SN	Themes	Sub-themes	Codes
1	Concerns regarding vaccine-induced infection	Ongoing concerns regarding HIV vaccine trials	A belief that HIV vaccine trial volunteers are infected with HIV
			Perceived side effects from the vaccine
		Reasons for ongoing concerns regarding HIV vaccine trials	Limited dissemination of information about HIV vaccine trials
			Existence of negative beliefs about vaccines
			Experience from previous medical experiments
		Anticipated reduction of concerns regarding HIV vaccine trial participation	Use of media to educate the community
			Dissemination of HIV vaccine trial information through former volunteers and flyers
2	Potential benefits of participation in HIV vaccine trials	Individual benefits of participation in HIV vaccine trials	Check-ups of health status
			Gaining knowledge regarding HIV vaccine trials
			Facilitation of access to medical services
		Perceived collective benefits of participation in HIV vaccine trials	Community protection
			Capacity building for local experts

### Theme one: Concerns regarding vaccine-induced infection

This theme illustrates about the suspicion of participants regarding becoming HIV infected through HIV vaccine trial participation and how to address these concerns. The section below provides details by sub-themes.

#### Sub-theme 1: Ongoing concerns regarding HIV vaccine trials

The participants expressed concern that the volunteers in the HIV vaccine trials became infected through participation in the trial. Non-HIV vaccine trial participants commonly expressed this type of doubt. Several participants believed that once a volunteer acquires HIV through the vaccine, he/she can get AIDS and cannot be cured. For that reason, they believed that the experimental vaccine was infectious. They said:

*Once infected with the virus, they [volunteers] will acquire AIDS straight away. If you have AIDS, you live with hope and eventually will die (FGD 5, non-HIV vaccine trial participant no.10)*.

Some non-HIV vaccine trial participants mentioned the consequences of being infected with HIV. They believed those who participated in the HIV vaccine trials were prone to harmful side- effects such as ill health leading to death. The participants who took part in the HIV vaccine trials did not believe that they could be infected by the experimental vaccine; however, they were concerned about the erroneous belief within the community that they were infected with viruses. They shared how the community perceived them:

*People said that all who participated [in the HIV vaccine trial] are victims. They believe those things [viruses] will erupt. Therefore, they are very observant that when it happens that we accidentally fall ill, maybe because we had not taken breakfast in the morning, they will say those things [viruses] have erupted … (FGD 3, HIV vaccine trial participant no. 4)*.

Another participant added:

*People do not have the correct information. For us who had participated; the community knows that we are infected with HIV (FGD 1, HIV vaccine trial participant no.9)*.

They stated that despite the erroneous belief from others that they had been infected with HIV; they strived to educate the community as expressed below:

*During HIVIS-03, many people were saying, ‘you are going to Muhimbili [trial site] to be infected with HIV, but we educated them…even the President would not accept his people to perish even if it is the police force (FGD 2, HIV vaccine trial participant, no*.*1)*

#### Sub-theme 2: Reasons for ongoing concerns regarding HIV vaccine trials

The participants reasoned that the ongoing concern surrounding HIV vaccine trials was fueled by inadequate information regarding the conduct of HIV vaccine trials in the respective communities. Those who took part in the previous HIV vaccine trials emphasized:

*The biggest problem is lack of education; they [people in the community] do not have adequate knowledge. They think once they join [take part in the vaccine trial], they will be infected with HIV viruses … the issue is knowledge" (FGD 6, HIV vaccine trial participant no. 9)*.

Several participants stated that the HIV vaccine trial researchers provided education, but that education did not adequately trickle into the surrounding community who perceived the trial as secret:

*Education [HIV vaccine trials information] did not reach all people. Therefore, those who did not get that education continue to spread incorrect information (FGD 3, HIV vaccine trial participant, no.5)*.

Other participants added:

*The vaccine research was not made public; it was like a secret; they did not announce … it was a secret in the sense that only a few people [volunteers] knew about it (FGD 4, HIV vaccine trial participant, no.1)*.

*For the first time when people heard about these trials, they did not get adequate time to understand… until now no results have been displayed publicly… Even myself that is my question until now (FGD 7, non-HIV vaccine trial participant, no*.*3)*

Additionally, the participants echoed that doubts within workplaces were fueled by the leaders, whom the participants trusted to be the most updated in regard to the HIV vaccine trials. They said that those leaders were not informed and that they discouraged volunteers instead of sharing facts to support the research. One participant lamented:

*Our leaders were not supportive… they were misleading us… they were telling us [volunteers] that, ‘so and so, there is a letter for you to go to be infected with the virus'… These are leaders; they must have been informed (FGD 2, HIV vaccine trial participant, no.5)*.

The participants reported that there was a breakdown in communication between the participating communities and trial researchers. They said such breakdown in communication encouraged rumors. Thus, feedback from trial researchers to the participating communities was demanded to provide clarification and to alleviate doubt. Nevertheless, unlike non-volunteers, it appeared that the volunteers accessed comprehensive information regarding the conduct of trials, and thus did not believe the surrounding rumors:

*After clarification, we accepted that the explanations [about vaccine trial] were adequate. The adequacy of information confirmed the truth… we are safe… truly I am healthy… this vaccine is safe (FGD 2, HIV vaccine trial participant, no.3)*.

The participants stated that negative beliefs regarding vaccines contributed to the doubt towards HIV vaccine trials that existed in the community. They believed that there was no difference between HIV and the HIV vaccine. This belief accelerated fears of acquiring incurable AIDS through the HIV vaccine study. Also, religious beliefs emerged as a controversial discourse regarding the HIV vaccine. Many believed that it was improper for a person to take part in an HIV vaccine study, as that person may have been involved in unsafe sexual practices. This could ultimately cause members of the community to associate the consequences of the unsafe sexual practices with the HIV vaccine. They said:

*Religion-wise, the disease itself is against morals [truthful life]. When a person appears for vaccination … it influences people to go against moral rules (FGD 7, non-HIV vaccine trial participant, no.3)*.

Another participant believed that HIV vaccines could affect the reproductive system:

*Vaccines cease family growth; I mean it decreases reproduction. In Tanzania there are some people when you talk about it [vaccine], they tell you that, ‘we cannot expose our bodies in experiments’ (FGD 1, HIV vaccine trial participant, no. 5)*.

Also, some participants were not in agreement with the medical experiments. They were concerned about past successes and failures of trials examining potential AIDS cures, as this is already a fatal disease. Alternatively, they believed that an imported vaccine would be good for Africans only if it had been tested on white people.

The participants reported that experiences from previous vaccine trials contributed to doubt regarding the experiments. They compared the HIV vaccine trial to a malaria vaccine trial. One participant narrated:

*I saw it [Malaria vaccine trial] in Ifakara [A research institute in Tanzania] how they did the trial. Those who participated were given the malaria vaccine after implanting malaria parasites. When the fever went up after implanting, they conducted the final stage of research (FGD 3, HIV vaccine trial participant, no.6)*.

Another participant added:

*Some soldiers took part in vaccine trials [unknown vaccine trial], but at some points; some experienced adverse effects … Previous trials have contributed to fear (FGD 2, HIV vaccine trial participant, no.3)*.

#### Sub-theme 3. Anticipated reduction of concerns regarding HIV vaccine trial participation

The participants commonly cited education in regard to HIV vaccine trials as an essential strategy that could contribute to the reduction in doubt among the community members. They emphasized that a comprehensive education should be provided to ensure understanding:

*People need to understand, and they have to be told that this vaccine is like this and this. Even us, we came to understand and agree because the explanations were satisfactory." (FGD 2, HIV vaccine trial participant, no. 3)*.

They suggested that for the HIV vaccine trial education to be effective, the provision of education should go beyond the study population. They said:

*Education should not be limited to volunteers. You need to prepare education through media, and you need to prepare programs either with ITV or TBC [local Television channels]… it should not only be limited to us volunteers because some of us are not able to spread the knowledge effectively" (FGD 2, HIV vaccine trial participant, no. 4)*.

They emphasized that volunteers should spend their time at both the workplace and in community, an arrangement that would offer them social support. Thus, the dissemination of information regarding HIV vaccine trials should be extensive. One volunteer said:

*I am not only living in the work environment; I have a family which follows behind and those who look at me with an evil eye. Lack of understanding extends beyond work environment (FGD 2, HIV vaccine trial participant, no.2)*.

Another participant emphasized his readiness to disseminate the information through the media:

*As a volunteer, I am ready to be on media like Clouds, ITV, so that they can interview me to explain how my life is after participation in the vaccine.' The people watching then may get knowledge, and even the misconceptions in their minds will decrease." (FGD 4, HIV vaccine trial participant, no. 2)*.

Some participants suggested that flyers and former HIV vaccine trial volunteers may be used to spread knowledge regarding HIV vaccine trials within the surrounding communities. They believed that flyers serve as useful educational materials. Also, they emphasized that former volunteers are the correct people to impart knowledge, and these volunteers may be impactful in disseminating the knowledge given their previous experience. One volunteer expressed:

*Let us use all means; flyers may not be adequate compared to using the volunteers to explain whatever they learned in practice including their current health status (FGD 1, HIV vaccine trial participant, no.3)*.

### Theme two: Potential benefits of participation in HIV vaccine trials

Despite the concerns surrounding HIV vaccine trials and anticipated solutions, several participants explained the advantages of participating in the previous HIV vaccine trials. The advantages ranged from individual to collective matters.

#### Sub-theme 4: Individual benefits of participation in HIV vaccine trials

The participants verbalized that participation in HIV vaccine trials improved their health status. The former volunteers appreciated the opportunity for pre-paid medical check-ups and access to treatment for various health problems. Most participants explained how this opportunity improved their health status and increased their willingness to access health services in the future. They said:

*“There were so many advantages because to participate in these studies [HIV vaccine trials], you first have to undergo a medical check-up. The act of being checked for your health status is one of the greatest benefits of participation. Another benefit is to know that your health is safe, and another benefit we got by participating was that for those of us who were found with some problems [unhealthy status], we were treated. Those were the benefits we got." (FGD 1, HIV vaccine trial participant, no. 1)*.

Additionally, the participants provided testimonies detailing the advantages gained by participation in the HIV vaccine studies. They said:

*I see that TAMOVAC gave me a new life because since I screened and got to know my health status … I was adequately educated about health matters. I saw it was important to start a new life (FGD 3, HIV vaccine trial participant, no.5)*.

*You will get to know your health status because, in the vaccine research, you meet many doctors who explain about your health. Also, you are taught how different diseases can be prevented and [receive] education on vaccines (FGD4, HIV vaccine trial participant, no.2)*.

*We were screened for various diseases, including cardiac, kidney, and liver. When you look at these diseases, it is expensive to screen. We were screened for free and provided with health insurance (FGD 4, HIV vaccine trial participant, no.1)*.

Some participants gained confidence in their health status after participating in HIV vaccine trials. They said they acquired adequate knowledge and had observed changes in their health-seeking behavior. They said:

*This research made me confident. For instance, in the past, I could have a health problem, but I never took it seriously. After enrolling in the research [HIV vaccine trial], the way they handled us, looking at our health status, it enhanced a positive perception about my health status. Even if I get flu, I will seek medical advice (FGD 3, HIV vaccine trial participant, no.2)*.

*I am happy since I participated in this TAMOVAC vaccine trial. I happen to be confident in the sense that even today if I sense any pain in my body, I am ready to go to the hospital to be screened and get treatment. In my life, I never thought of getting screening for the whole system of my body (FGD 3, HIV vaccine trial participant, no.3)*.

The participants stated that after participating in the HIV vaccine trials, they gained knowledge and the courage to disseminate facts about HIV matters. Also, this gained knowledge enabled them to convince others to undergo health check-ups. One volunteer said:

*I can stand before the public to educate and convince people about the HIV test and understand. In short, I am grateful that they [trial team] had given me the courage to stand anywhere and explain to people about the importance of their health and the HIV test." (FGD 3, HIV vaccine trial participant, no. 6)*.

Through HIV vaccine trials, some participants were able to establish new networks with the research team. In turn, these networks became the entry to medical services when they encountered health related problems. Thus, participation in the HIV vaccine trials was an opportunity to enlarge their social networks. These networks were useful in a practical manner, as volunteers used them to fast-track access to appropriate medical consultations. One volunteer explained how he received assistance at Muhimbili National Hospital as a result of the secured network with the vaccine trial doctors:

*I once had a boy of mine who was made blind and had his bowels out. … I remembered Dr. Y [trial research Doctor] and called him and told him I was stuck somewhere. You see the relationship after getting in the trial, and it is like an asset than any other thing" (FGD 3, HIV vaccine trial participant, no. 4)*.

#### Sub-theme 5: Perceived collective benefits of participation in HIV vaccine trials

The participants perceived that participation in HIV vaccine trials would save the lives of the new generation by preventing new HIV infections. They said:

*After completion of the trial, we expect that if the vaccine is successfully developed, then it will help not only us but the next generation (FGD 4, HIV vaccine trial participant, no.1)*.

*Our society will benefit through us from the fact that by the end*, *the medicine [vaccine] will benefit all people*, *and there will be no doubt or fear because we have sacrificed… we get the benefit … not only for our nation but the world (FGD 2*, *HIV vaccine* trial *participant*, *no*.*3)*.

The participants believed that through the participation in HIV vaccine trials, the availability of preventive HIV vaccines would also decrease the number of orphans and street children. One youth said:

*If we eliminate this disease, I believe we Tanzanians will overcome this disease … we can also reduce the number of orphans and street children because if you give the vaccine to the mother or a person, it will be very difficult to acquire the disease [AIDS] in the future. (FGD 8, non-HIV vaccine trial participant, no. 6)*.

Many participants believed that engagement in HIV vaccine trials provided an opportunity among the local scientists to build the capacity of conducting HIV vaccine research. They believed that researchers who engage in the HIV vaccine trials contributed great benefits to scientific investigations and complemented the efforts of searching for an HIV vaccine:

*It [vaccine trial research] helps our Tanzanian researchers to gain experience of doing various studies, HIV vaccine being among them…thus it advances skills and capacity among our health experts (FGD 2, HIV vaccine trial participant, no.4)*.

The participants emphasized that through HIV vaccine trial research, Tanzania will gain international recognition. They envisioned Tanzanian scientists as existing among the international scientists who worked tirelessly to search for a vaccine against HIV infection. One youth narrated:

*We [Tanzanians]will be more recognized internationally because in 24 hours scientists are scratching their heads looking for cure of this disease [AIDS]… if we manage we will largely make a very big progress and will have that sense of confidence because will have done a big thing [contribution in HIV vaccine development].” (FGD IDC non-HIV vaccine trial participant, no. 6)*.

Overall, these findings enhance the understanding of community perceptions regarding the implementation of HIV vaccine trials in a low-income setting.

## Discussion

Overall, the results of this study demonstrate the importance of adequately educating the community on matters underlying HIV vaccine trials. It was clear that varied opinions existed between those who participated in the trials and those who did not. Doubts towards HIV vaccine trials were primarily reported among participants who did not participate in the HIV vaccine trial. Similarly, concerns of acquiring HIV infection from the vaccine and perceived side effects that were attributed to a lack of understanding of HIV vaccine trials, and the existence of negative beliefs regarding vaccines and bad experiences from previous medical experiments were primarily voiced by HIV vaccine trial non-participants. The use of media, former volunteers in HIV vaccine trials, and flyers are envisioned as strategies to disseminate facts regarding HIV vaccine trials. In contrast, it was reported by most participants in the trials that participation in HIV vaccine trials resulted in positive outcomes at the individual and community levels. Health check-ups, increased knowledge regarding HIV vaccine trials, and the establishment of new social networks are considered to be individual gains. Additionally, community protection from HIV infection and capacity building among local experts both underscore the perceived community benefits of participation in HIV vaccine trials.

The fact that concerns regarding HIV vaccine trials emerged among participating communities implies limited understanding of experimental HIV studies. Mistrust from the surrounding communities towards the HIV vaccine trials was one of the concerns that were identified during the prolonged follow up of HIVIS-03 volunteers; however, this mistrust decreased over time [27]. Also, in the present study erroneous beliefs and perceived bad experiences from previous medical experiments may have fueled the concerns. Additionally, fear of vaccine-induced HIV seropositivity [20, 24, 33–34] has been reported elsewhere, and this influenced participation in HIV vaccine trials. Increasingly, previous studies revealed a demand for basic HIV vaccine education to address vaccine trial-related concepts [12–16, 35].

To understand the implementation of HIV vaccine trials in various contexts, HIV vaccine knowledge is needed. The present study provides suggestions that can be used to widen the dissemination of HIV vaccine trial information beyond trial participants. To promote future participation in HIV vaccine trials, locally suggested strategies are needed to address these concerns. The suggested use of media and former volunteers to halt the doubts surrounding HIV vaccine studies in the respective communities may represent an alternative means to disseminate the facts in public. In a previous study, social marketing was predicted to increase future HIV vaccine uptake and clinical trial participation [36]. Also, tailored interventions to address concerns related to HIV vaccine trials have been proposed in other studies [37–38]. Even though the participating communities were well prepared for the phase I/II HIV vaccine trials, doubts still emerged within these communities regarding the conduct of those trials. Active community engagement in HIV vaccine trials was an essential strategy to maximize understanding of the study. A literature review suggests the need to monitor and evaluate the impact of various community engagement strategies on the implementation of HIV trials and the uptake of products [17].

Participation in HIV vaccine trials heavily depends upon the understanding of trial concepts. In the present study, the volunteers who fully participated and completed the Phase I/II HIV vaccine trials did not express doubt. The lack of doubt among these volunteers was possibly due to acquired knowledge and time to absorb the knowledge through a series of workshops and follow up visits [26]. Similarly, Mutua G *et al*. [39] found that low risk volunteers who were followed up to 16 months in Kenya, Uganda, Rwanda, Zambia, and South Africa reported positive social impacts and few significant negative impacts after participation in phase I HIV vaccine trials. Repeated engagement with trial staff and time to absorb the information may be the cornerstone required to address the information shortfalls in HIV vaccine trials. Ott and colleagues [40] recommend education regarding basic concepts related to clinical trials, time to absorb materials, and assessment of understanding in future biomedical prevention trials. Additionally, community involvement in the vaccine trial process appears essential and can be improved through increased education and capacity building, education of the trial participants, use of community advisory boards, and partnership development [41].

The expressed benefits from participants in HIV vaccine trials are noteworthy. The health check-ups and medical services available to trial participants were valued by participants. In HIVIS-03, the potential study participants envisioned free health services as a great benefit if they participate in HIV vaccine trials [24]. Additionally, the eventual trial volunteers appreciated the free health check-ups as one of the advantages they gained during the HIV vaccine trials [26]. A literature review recommends long-term follow-up studies of future vaccine study participants in HIV vaccine trials to monitor the potential benefits and harms associated with participating in HIV vaccine trials and to better understand the triggers of social harm [17].

### Limitations

The study was conducted using an open cohort of youths, police, and prison officers. The UNAIDS Good Participatory Practice Guidelines for community engagement in the conduct of biomedical research were adhered to throughout the HIV vaccine trial periods; however, the nature of work, particularly among the police and prison officers, required frequent job relocations. Thus, although education sessions to engage the community were performed previously, during the trials some officers may have been subjected to relocation during the trial period. This job relocation may have influenced the findings.

These qualitative findings are therefore limited to the studied community; however, they can be transferred to similar contexts. Also, the knowledge generated may improve information dissemination in future trials. Further research is necessary to quantify these results.

## Conclusions

The perceptions within participating communities post-Phase I/II HIV vaccine trials are noteworthy. The findings highlight the dissimilarity in the understanding of HIV vaccine trials between those who participated and those who did not. Unlike the volunteers who fully participated in the HIV vaccine trials, the observed concerns among those who did not may be due to the lack of comprehensive knowledge regarding HIV vaccine trials. The conduct of future HIV vaccine trials requires wider community dissemination of information to increase the understanding of HIV vaccine studies. Also, to ensure timely dissemination of the respective trials, the trial researchers should provide regular feedback to the public and to the communities surrounding the trial participants.
